# Cytopenia after chimeric antigen receptor T cell immunotherapy in relapsed or refractory lymphoma

**DOI:** 10.3389/fimmu.2022.997589

**Published:** 2022-09-05

**Authors:** Jin Zhou, Ying Zhang, Meng Shan, Xiangping Zong, Hongzhi Geng, Jiaqi Li, Guanghua Chen, Lei Yu, Yang Xu, Caixia Li, Depei Wu

**Affiliations:** ^1^ National Clinical Research Center for Hematologic Diseases, Jiangsu Institute of Hematology, The First Affiliated Hospital of Soochow University, Suzhou, China; ^2^ Institute of Blood and Marrow Transplantation, Collaborative Innovation Center of Hematology, Soochow University, Suzhou, China; ^3^ Key Laboratory of Stem Cells and Biomedical Materials of Jiangsu Province and Chinese Ministry of Science and Technology, Suzhou, China; ^4^ Institute of Biomedical Engineering and Technology, Shanghai Engineering Research Center of Molecular Therapeutics and New Drug Development, School of Chemistry and Molecular Engineering, East China Normal University, Shanghai, China

**Keywords:** Lymphoma, chimeric antigen receptor, hematological toxicity, cytopenia, cytokine release syndrome

## Abstract

**Background:**

Patients with relapsed or refractory (R/R) lymphomas have benefited from chimeric antigen receptor (CAR)-T-cell therapy. However, this treatment is linked to a high frequency of adverse events (AEs), such as cytokine release syndrome (CRS), immune effector cell-associated neurotoxicity syndrome (ICANS), and hematologic toxicity. There has been increasing interest in hematological toxicity in recent years, as it can result in additional complications, such as infection or hemorrhage, which remain intractable.

**Methods:**

We conducted a retrospective, single-institution study to evaluate the patterns and outcomes of cytopenia following CAR-T-cell infusion and potential associated factors.

**Results:**

Overall, 133 patients with R/R lymphoma who received CAR-T-cell therapy from June, 2017 to April, 2022 were included in this analysis. Severe neutropenia, anemia and thrombocytopenia occurred frequently (71, 30 and 41%, respectively) after CAR-T-cell infusion. A total of 98% of severe neutropenia and all severe thrombocytopenia cases occurred in the early phase. Early severe cytopenia was associated with CRS incidence and severity, as well as peak inflammatory factor (IL-6, C-reactive protein (CRP), and ferritin) levels. In multivariate analysis, prior hematopoietic stem cell transplantation (HSCT), baseline hemoglobin (HB), and lymphodepleting chemotherapy were independent adverse factors associated with early severe cytopenia. In addition, 18% and 35% of patients had late neutrophil- and platelet (PLT)-related toxicity, respectively. In multivariate analysis, lower baseline PLT count was an independent factor associated with late thrombocytopenia. More severe cytopenia was associated with higher infection rates and poorer survival.

**Conclusions:**

This research indicates that improved selection of patients and management of CRS may help to decrease the severity of cytopenias and associated AEs and improve survival following CAR-T-cell therapy.

**Clinical Trial Registration:**

https://www.clinicaltrials.gov/ct2/show/NCT03196830, identifier NCT03196830.

## Introduction

Although chemoimmunotherapy regimens and novel targeted drugs have substantially improved lymphoma care (1), patients with treatment failure after chemoimmunotherapy often have a poor outcome, especially those with disease that is refractory to frontline or subsequent therapies (2). Chimeric antigen receptor (CAR)-T-cell therapy is a novel type of tumor immunotherapy that has improved outcomes for many patients with relapsed or refractory (R/R) lymphomas, showing varied efficacy in diffuse large B-cell lymphoma (DLBCL) (objective response rate (ORR) 52-88%; complete response (CR) 40-59% (3), mantle cell lymphoma (MCL) (CR 59%) (4), and chronic lymphocytic leukemia (CLL) (ORR 57-74%; CR 21%) (5). To date, several CAR-T-cell therapies have received FDA approval in the USA, including axicabtagene ciloleucel (axi-cel) (6) and lisocabtagene maraleucel (liso-cel) (7) for R/R DLBCL, axi-cel for R/R follicular lymphoma (FL) (8), and brexucabtagene autoleucel (brexu-cel) for R/R MCL and B-cell acute lymphoblastic leukemia (ALL) in adults (4). CAR-T-cell therapy has had a substantial impact on overall survival (OS) and progression-free survival (PFS), but it also comes with a host of adverse events (AEs), including cytokine release syndrome (CRS) and immune effector cell-associated neurotoxicity syndrome (ICANS), which can lead to treatment failure (9). Increasing CAR-T-cell therapy experience suggests that these complications can be effectively controlled in a timely manner through early monitoring and intervention, which makes this treatment safer than ever (7).

However, with long-term patient follow-up, cytopenia has been increasingly reported after CAR-T-cell therapy. Cytopenia after infusion of CAR-T cells is extremely common. In the ZUMA-1 study, 78% of patients had neutropenia, 43% of patients had anemia and 38% of patients had thrombocytopenia (10). Similarly, any-grade cytopenia occurred in 44% of patients in the JULIET study (11). Severe cytopenia may result in higher risks of infection (12) and hemorrhage, requiring more blood transfusion support (13). Transfusion-related responses, iron overload, and circulatory overload are relevant to continuous transfusion needs. As a result, there might be a decline in quality of life, and an increase in treatment-related morbidity and mortality (14). Prior studies have mainly considered the following potential risk factors: patient median age, number of prior lines of therapy, baseline blood counts, baseline LDH levels, CRS grade, and CRS-related inflammatory factor levels (14–16). However, these factors have shown contradictory utility in identifying and predicting the risk of this complication. Here, we retrospectively characterized the hematological toxicities of patients treated in our center who participated in a phase I/II clinical trial of CAR-T-cell therapy for R/R lymphoma and explored potential causes of hematologic toxicities, specifically cytopenias, with CAR-T-cell therapy.

## Methods

### Patients

**Figure 1:**
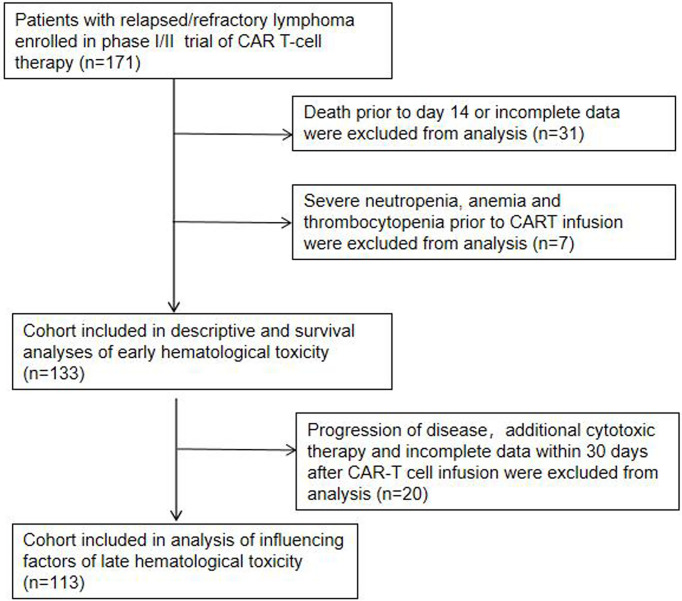
Patient selection.

In this single institution retrospective observational study, 171 patients with R/R lymphoma, including DLBCL, transformed lymphoma (transformed FL (tFL), transformed mucosa-associated lymphoid tissue (tMALT) lymphoma, or transformed CLL (tCLL)), indolent lymphoma (marginal zone lymphoma (MZL), CLL, or FL) and other diseases (**Table 1**), were treated with CAR-T cells targeting CD19/CD22/CD20/CD30 between June, 2017 and April, 2022 (NCT03196830) at our center. We analyzed 133 patients without severe cytopenia before reinfusion, death prior to d14 or incomplete data for early hematologic toxicity within one month after infusion. 113 patients without disease progression, additional cytotoxic therapy or incomplete data were further analyzed for late hematologic toxicity at one month after infusion (**Figure 1**). This study was approved by the Institutional Ethics Committee of the First Affiliated Hospital of Suzhou University and was in line with the guidelines of the Declaration of Helsinki.

**Table 1:** Demographic and clinical characteristics of patients.

Total	N=133
Age, year	44 (range, 18-71)
Sex, male n (%)	75 (56.39%)
Bone marrow involvement n (%)	45 (33.83%)
Prior HSCT n (%)	24 (18.05%)
Median lines of prior therapy (range)	2 (range, 1-7)
**CAR Product **	
CD19 n (%)	29 (21.80%)
CD19/CD22 n (%)	84 (63.16%)
CD19, CD20 n (%)	12 (9.02%)
CD30 n (%)	8 (6.01%)
**Disease Entity **	
DLBCL n (%)	87 (65.41%)
PMBCL n (%)	5 (3.76%)
GZL n (%)	2 (1.50%)
HL n (%)	3 (2.26%)
Transformed Lymphoma (trFL, trMCL, trMZL, trCLL) n (%)	17 (12.78%)
Indolent lymphoma (MZL, CLL, FL) n (%)	6 (4.51%)
AITL n (%)	3 (2.26%)
MCL n (%)	5 (3.76%)
BL n (%)	5 (3.76%)
**Pre-lymphodepletion**	
Median NE, ×10^9^/L	1.91 (0.56-28.77)
Median PLT, ×10^9^/L	104 (20-776)
Median HB, g/L	89.5 (61-179)
Median LDH, U/L	237.15 (115.6-11637)
Median CRP, mg/L	36.73 (1.04-182.02)
Median Ferritin, ng/mL	520.10 (17.56-8969.29)
**Lymphodepleting preparative regimen**	
FC n (%)	120 (90.22%)
SEAM n (%)	13 (9.77%)
**CRS, n (%) **	
Grade 0	46 (33.08%)
Grade 1-2	67 (50.38%)
Grade 3-5	20 (15.03)
Neurotoxicity, n (%)	9 (6.43%)

Data were described as n (%) or median [range].

HSCT, hematopoietic stem cell transplantation, DLBLC, Diffuse Large B-Cell Lymphoma, PMBCL, Primary Mediastinal B-Cell Lymphoma, GZL, Gray zone lymphoma, HL, Hodgkin lymphoma, FL, Follicular lymphoma, MCL, Mantle cell lymphoma, MZL, Marginal zone lymphoma, CLL, Chronic lymphocytic leukemia, AITL, Angioimmunoblastic lymphoma, BL, Burkitt lymphoma, NE, neutrophil, PLT, Platelet, HB, Hemoglobin, LDH, lactate dehydrogenase, CRP, C reactive protein, FC, fludarabine 30 mg/m2 d-5 to -3, cyclophosphamide 300 mg/m2 d-5 to -3, SEAM: 250 mg/m2 Me-CCNU d-10, etoposide 100 mg/m2 every 12h d−9 to−6, cytarabine every 12 h d−9 to-6, hematopoietic stem cell infusion d-2.

### Study design

Patients were assessed for eligibility and their peripheral blood mononuclear cells (PBMCs) were obtained for CAR-T-cell production. The costimulatory domain of all CARs was 4-1BB. Prior to this report, our center reported the protocol for manufacturing CAR-T cells and the construct designs for the CARs (17). Patients received lymphodepleting chemotherapy with FC (fludarabine 30 mg/m^2^ d-5 to d-3, cyclophosphamide 300 mg/m^2^ d-5 to d-3) or SEAM (250 mg/m^2^ Me-CCNU d-10, etoposide 100 mg/m^2^ every 12 h d-9 to d-6, cytarabine every 12 h d-9 to d-6, hematopoietic stem cell infusion d-2). A total of 1×10^7^ CAR-T-cells/kg were infused over 3 consecutive days (d0, 10%; d1, 30%; d2, 60%). Subsequently, the hospital observation period was at least two weeks, and the period was extended according to whether the patient developed AEs. Regular outpatient follow-up monitoring was performed after the patients were discharged from the hospital for one month. In addition to routine blood tests, the patients’ cytokine, serum ferritin, and C-reactive protein (CRP) levels were also regularly monitored.

### Definitions of hematologic toxicity

We defined neutropenia, anemia, and thrombocytopenia using guidelines provided by the Center for International Blood and Marrow Transplant Research (CIBMTR). The definitions of neutropenia and severe neutropenia were an absolute neutrophil count below 1.5×10^9^/L and 0.5×10^9^/L peripheral blood, respectively. Anemia was defined as a hemoglobin (HB) concentration less than 120 g/L in men and less than 110 g/L in women; severe anemia was defined as an HB concentration lower than 60 g/L. Platelet (PLT) counts below 100×10^9^/L and below 20×10^9^/L were used to characterize thrombocytopenia and severe thrombocytopenia, respectively. Severe cytopenia was defined as any occurrence of severe neutropenia, anemia or thrombocytopenia. Early hematologic toxicity was defined as cytopenia that occurred within one month, and late hematologic toxicity was defined as neutrophil count lower than 1.0×10^9^/L or PLT count lower than 80×10^9^/L at one month after infusion. The primary observational endpoint of the study was the severity of hematological toxicity up to 30 days after CAR-T-cell infusion, and the secondary observational endpoint was neutrophil- and PLT-related toxicity 30 days after CAR-T-cell infusion.

### CRS, ICANS, serum biomarkers, cytokines and additional clinical characteristics

Experienced doctors evaluated CRS and ICANS in accordance with the criteria agreed upon by the American Society for Transplantation and Cellular Therapy (ASTCT) (18). Patient serum was examined for the levels of IL-2, IL-4, IL-6, IL-10, IL-17, IFNγ, TNF,CRP, and ferritin. We also closely monitored patient vital signs and complications, such as infection and hemorrhage. In addition, patient clinical outcomes were determined.

### Statistics

The baseline features and cytopenia statuses of the patients were described using descriptive statistics. For the purpose of determining statistical significance (p<0.05) between groups, a nonparametric Mann-Whitney test was performed. Categorical variables were analyzed with the chi-square test. Spearman correlation coefficients were used to evaluate associations between continuous variables. OS data were displayed using Kaplan-Meier curves. Independent predictors of cytopenia were assessed by univariate and multivariate logistic regression analyses. IBM SPSS Statistics version 26 and GraphPad Prism v9.0 were used to conduct the statistical analysis.

## Results

### Patient characteristics

Between June, 2017 and April, 2022, 171 patients with R/R lymphoma were treated with CAR-T-cells. A total of 133 patients who survived for more than 14 days and had complete data were included in this analysis. The median age was 44 years (range, 18-71). Forty-five (34%) patients had bone marrow involvement at diagnosis. Patients received a median of 2 prior therapies (range, 1-7), and 24 (18%) had HSCT before the lymphodepletion regimen. Twenty-nine (21%) patients were infused with CD19 CAR-T-cells, 84 (63%) patients were treated with CD19/CD22 bispecific CAR-T-cells, 12 (9%) patients were sequentially infused with CD19 and CD20 CAR-T-cells, and eight (6%) were treated with CD30 CAR-T-cells. The main indications for CAR-T-cell therapy were R/R DLBCL (n=87), transformed B-cell lymphoma (n=17), indolent lymphoma (n=6), and primary mediastinal large B-cell lymphoma (PMBCL) (n=5). Eighty-seven (65%) patients presented with CRS after CAR-T-cell infusion, and ICANS occurred in 9 (6%) patients. A total of 31 (22%) patients received steroids or tocilizumab to cure severe (grade ≥2) CRS and ICANS. The patient characteristics are depicted in **Table 1**.

### Hematologic toxicity after CAR-T-cell Infusion

**Figure 2:**
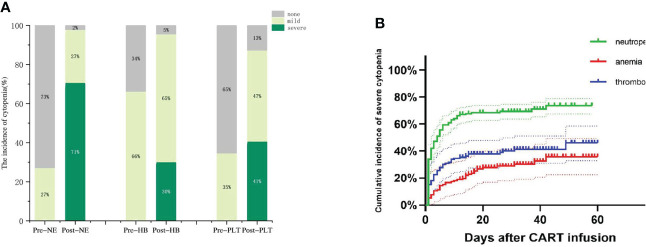
Hematologic toxicity after CAR-T cells. **(A)**The incidence of cytopenia before and after CAR-T cell infusion. **(B)** Cumulative incidence of severe cytopenia.

We observed that prior to lymphodepleting chemotherapy, the median neutrophil count was 1.96 (range, 0.56-28.77) ×10^9^/L, the median HB concentration was 89.5 (range, 61-179) g/L, and the median PLT count was 104 (range, 20-776) ×10^9^/L (**Table 1**). After CAR-T-cell infusion, 130 (98%) patients developed neutropenia, 71% of them developed severe neutropenia, and 30% and 41% of patients developed severe anemia and thrombocytopenia, respectively (**Figure 2A**).

Next, we observed three lineages kinetics after CAR-T-cell infusion. The median time to severe neutropenia was 4 days from cell infusion (range, 1-58), and the cumulative incidence of severe neutropenia on d30 was 69.45% (95% CI, 64.32%-75.34%). The median time to severe anemia and thrombocytopenia was not reached, and the cumulative incidence of severe anemia and thrombocytopenia on d30 after CAR-T-cell infusion was 39.88% (95% CI, 29.92%-50.04%) and 28.82% (95% CI, 16.88%-39.81%), respectively. Compared to severe neutropenia, severe anemia and thrombocytopenia occurred more slowly (**Figure 2B**).

### Early hematological toxicity

We considered that lymphodepleting chemotherapy may induce cytopenia, which generally occurs within 3-4 weeks after chemotherapy. Apart from this, we explored other factors that may be associated with the onset of early severe cytopenias. Based on the data of 133 patients without severe cytopenia before reinfusion, death prior to d14 and incomplete data within one month after infusion, 98% of severe neutropenia cases and all severe thrombocytopenia cases occurred in the early phase.

#### CRS and inflammatory cytokine levels correlate with the occurrence of early severe hematologic toxicity

**Figure 3:**
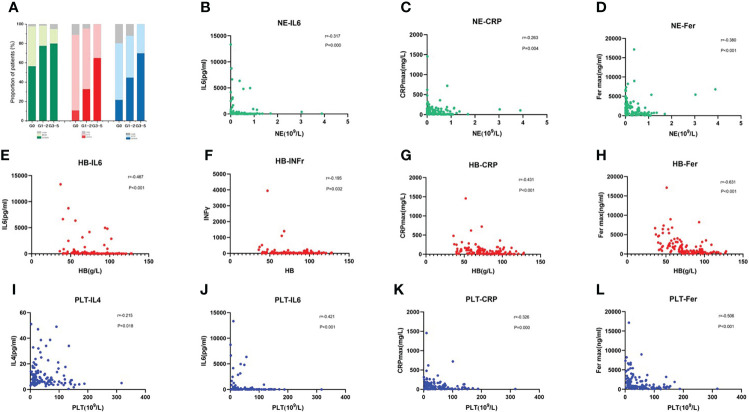
CRS and Inflammatory Cytokine Levels Correlate with the Occurrence of Early Severe Haematologic Toxicity **(A)** Incidence of each cytopenia is shown in patients without CRS or mild or severe CRS. **(B–L)** Correlation of inflammatory factors and blood cell parameters, including neutrophil count **(B–D)**, hemoglobin concentration **(E–H)**, and platelet count **(I–L)**. P values and r values were determined by Spearman correlation analysis.

CRS was the most frequent AE recorded in patients receiving CAR-T-cell treatment. We found that as the CRS developed more severe, the incidence of each degree of cytopenia tended to increase accordingly (**Figure 3A**). We further analyzed the correlation between the minimum blood cell count and maximum inflammatory factor levels related to CRS in the early phase. The peak IL-6, CRP, and ferritin levels were substantially inversely related to the neutrophil, HB, and PLT counts. Furthermore, the peak value of IFNγ was negatively correlated with the minimum HB value, and the peak IL-4 level was negatively correlated with the minimum PLT count (**Figures 3B–L**).

#### Factors associated with the occurrence of early severe cytopenia

**Figure 4:**
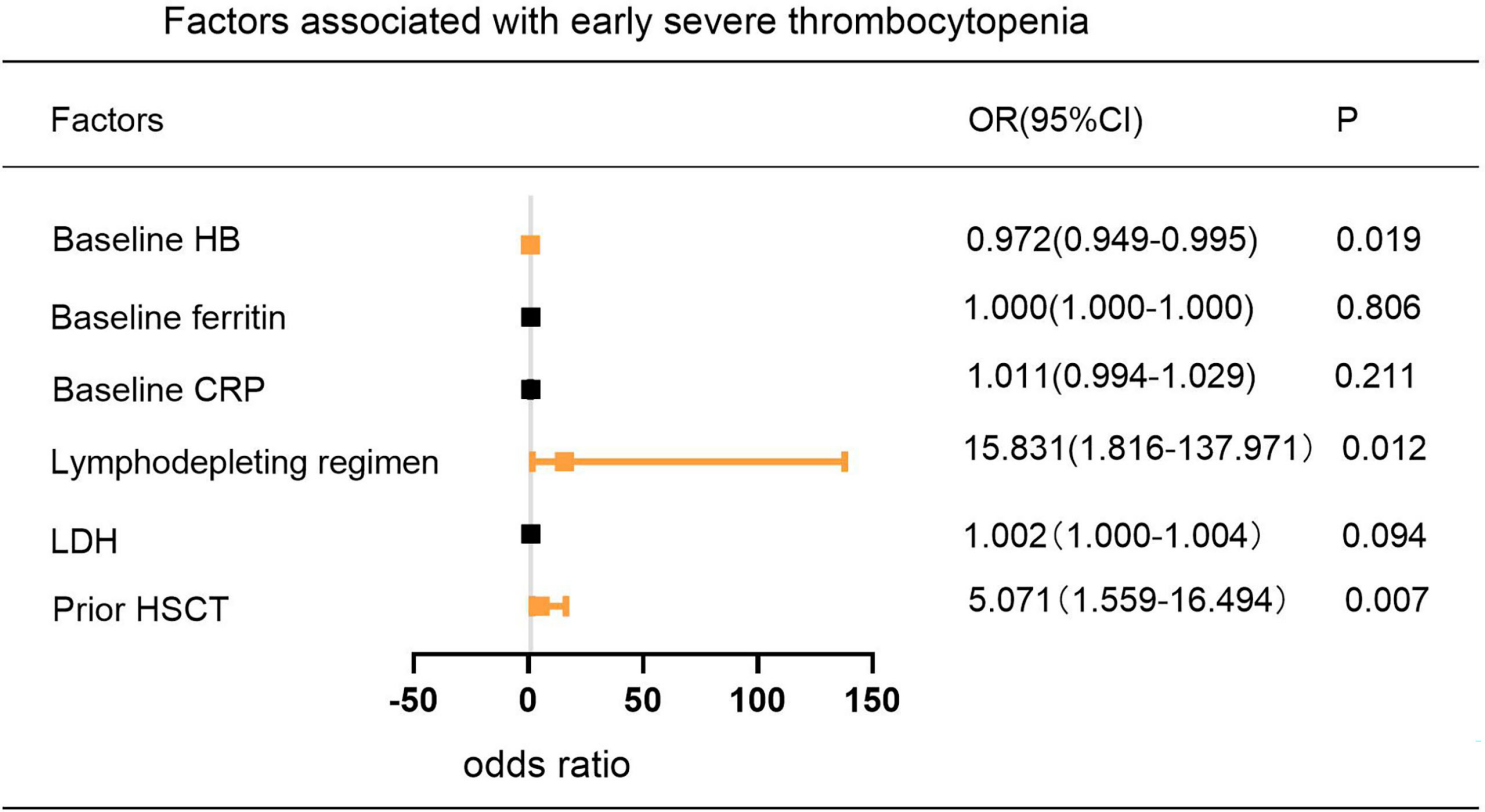
Multivariable Analysis of Factors Associated with the Incidence of Early Severe Thrombocytopenia. P values were tested by logistic regression model.

According to previous studies (16, 19), 14 baseline clinical characteristics considered to have potential prognostic value for predicting severe cytopenia are age, sex, disease type, tumor stage (≤II/>II), bone marrow involvement (yes/no), lines of prior therapy (≤2/>2), response to pre-CAR-T-cell treatment (partial response (PR)/stable disease (SD), progressive disease (PD)), prior HSCT (yes/no), LDH, lymphodepleting chemotherapy (FC/SEAM), CAR product, baseline blood parameters, baseline CRP and baseline ferritin. In multivariate analysis, prior HSCT and baseline HB were independent adverse factors associated with the occurrence of early severe neutropenia and thrombocytopenia. In addition, lymphodepleting chemotherapy with SEAM was another independent adverse factor associated with the occurrence of early severe thrombocytopenia (**Figure 4**) (**Supplemental Tables 1–3**).

#### Complications, treatments and prognoses associated with early severe cytopenia

We analyzed the correlation between severe cytopenia and early CAR-T-cell infusion outcomes. Patients with severe cytopenia had a significantly increased infection rate. (P=0.001). In the subgroup that received broad-spectrum carbapenem and tigecycline antibiotics, the number of patients with severe cytopenia was significantly higher than the number of patients with non-severe cytopenia (P=0.000). Although there were no significant differences in hemorrhage rate (P=0.328), patients with severe cytopenia received significantly more blood transfusions than patients without severe cytopenia (P<0.001) (**Table 2**).

**Table 2:** Early severe hematological toxicities with cost of treatment and other adverse events after CAR-T-cell infusion.

Characteristic	Severe cytopenia	Non-Severe cytopenia	p
	n=99	n=34	
Infection, n (%)	32 (94.11%)	2 (5.88%)	0.001
Number of patients using broader-spectrum antibiotic, n (%)	73 (87.95%)	10 (12.05%)	0.000
Hemorrhage, n (%)	5 (100%)	0 (0.00%)	0.328
Number of blood transfusions, n (%)	7.05 (0-52)	0.76 (0-17)	<0.001

Data were described as n (%).

P value were tested by Chi-Square test.

**Figure 5:**
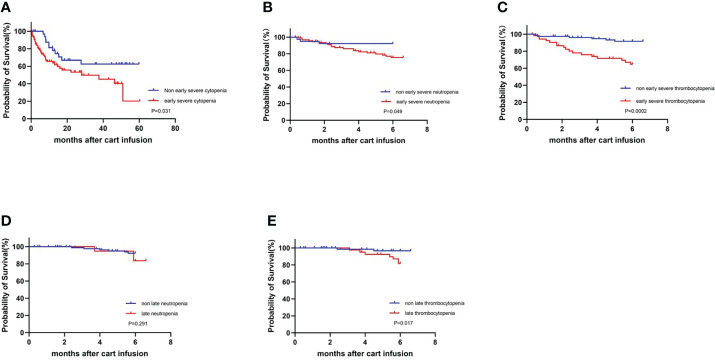
Survival curves for all patients who underwent infusion of CART cell with or without severe cytopenia. KM estimates of OS **(A)** in patients with non-severe cytopenia (blue) and with severe cytopenia (red). **(B, C)** Early severe neutropenia and thrombocytopenia compared with non-occurring group at 6-month survival.**(D, E)** Late neutropenia and thrombocytopenia compared with non-occurring group at 6-month survival.

In addition, 133 patients were followed up until April 1, 2022, and the survival curves are shown in **Figure 5A**. The median follow-up time was 10.2 months (range, 0.3 to 60.3 months), and compared with severe cytopenia patients, patients with non-severe cytopenia experienced significantly prolonged survival (P=0.031). We further analyzed the association of early severe neutropenia and thrombocytopenia with the occurrence of mortality 6 months post CAR-T-cell therapy. The 6-month survival rate of patients in the early severe neutropenia and thrombocytopenia group was significantly lower than that in the other group (P=0.049, P=0.000) (**Figures 5B, C**).

#### Late hematological toxicity

Cytopenia after 30 days is considered as late hematologic toxicity. We further analyzed the 113 patients without progression of disease, additional cytotoxic therapy and incomplete data for late hematologic toxicity at one month after infusion. Thirty days after CAR-T-cell infusion, 20 (18%) patients had neutrophil count lower than 1.0×10^9^/L, and 39 (35%) patients had PLT count lower than 80×10^9^/L. Similarly, we analyzed the association between late cytopenia and survival. There was no statistically significant difference in 6-month survival between patients who had late neutropenia and no neutropenia (P=0.291), but compared with the late thrombocytopenia group, patients without late thrombocytopenia had a significantly prolonged 6-month survival (P=0.017) (**Figures 5D, E**).

#### Factors associated with late hematological toxicity

We also included early CRS and inflammatory factor variables as well as clinical baseline characteristics to explore potential factors associated with late hematologic toxicity. In univariate analysis, prior HSCT, lower baseline HB/PLT, higher baseline ferritin and ferritin peak after infusion were associated with late thrombocytopenia, while no factor was found to be significantly associated with late neutropenia (**Supplement Table 4**). In multivariate analysis, lower baseline PLT was an independent adverse factor associated with late thrombocytopenia (**Table 3**).

**Table 3:** Univariate and Multivariable Analysis of Factors Associated with the late neutropenia.

Variable	Univariable		Multivariable	
	Hazard ratio (95%CI)	P	Hazard ratio (95%CI)	P
Age	0.993 (0.962-1.024)	0.650		
Sex (M/F)	0.481 (0.218-1.059)	0.069		
Disease Type				
Aggressive B-cell lymphoma^a^	1^c^			
Indolent B-cell lymphoma^b^	8.567E+8 (0.000-)	0.999		
AITL	8.077E+8 (0.000-)	0.999		
HL	2.610E+18 (0.000-)	0.999		
Ann Arbor Stage (≤II/>II)	1.230 (0.353-4.285)	0.745		
Bone marrow involvement (yes/no)	0.852 (0.376-1.930)	0.701		
lines of prior therapy (≤2/>2)	1.561 (0.711-3.425)	0.267		
Prior HSCT (yes/no)	2.818 (1.118-7.102)	0.028	2.300 (0.742-7.126)	0.149
Response before treatment (PR/SD, PD)	1.318 (0.591-2.940)	0.500		
Pre-LD NE	0.985 (0.879-1.105)	0.801		
Pre-LD HB	0.960 (0.938-0.982)	0.000	0.980 (0.955-1.006)	0.140
Pre-LD PLT	0.985 (0.977-0.992)	0.000	0.989 (0.980-0.997)	0.006
LDH	1.001 (0.999-1.003)	0.245		
Lymphodepleting chemotherapy (FC/SEAM)	1.195 (0.363-3.935)	0.770		
CAR Product				
CD19	1^d^			
CD19/CD22	1.385 (0.207-9.241)	0.737		
CD19, CD20	0.920 (0.157-5.389)	0.926		
CD30	1.667 (0.210-13.223)	0.629		
Baseline CRP	1.013 (0.998-1.029)	0.093		
Baseline ferritin	1.001 (1.000-1.001)	0.024	1.000 (0.999-1.001)	0.834
CRS grade (<2/≥2)	0.850 (0.329-2.194)	0.737		
CRP max	0.999 (0.997-1.002)	0.613		
Ferritin max	1.000 (1.000-1.001)	0.015	1.000 (1.000-1.001)	0.294
IL2 max	0.974 (0.933-1.016)	0.219		
IL4 max	0.952 (0.906-1.000)	0.050		
IL6 max	1.000 (1.000-1.000)	0.974		
IL10 max	0.999 (0.993-1.004)	0.603		
TNFmax	0.996 (0.976-1.017)	0.707		
IFNγ max	0.997 (0.991-1.003)	0.339		
IL17 max	0.991 (0.977-1.006)	0.232		

Aggressive B-cell lymphoma^a^ include DLBCL, PMBCL, GZL, Transformed Lymphoma, MCL, BL.

Indolent B-cell lymphoma^b^ include MZL, CLL, FL.

Aggressive B-cell lymphoma^c^ group was defined as the control group.

CD19^d^ group was defined as the control group.

## Discussion

CAR-T-cell therapy has been shown to provide long-term remission or cure for patients with R/R lymphoma. However, there is a significant incidence of AEs with this medication, including CRS, ICANS, and hematologic toxicity. CAR-T-cell related CRS and neurotoxicity have been widely reported, and grading systems and care guidelines developed. However, hematologic toxicity following CAR-T-cell treatment for lymphoma remains poorly characterized. This retrospective review details the early and late hematologic toxicity patterns seen at our institution in a sizable cohort of R/R lymphoma patients who received CAR-T-cell treatment.

After CAR-T-cell infusion, we observed a high incidence of cytopenia, almost all of our patients had at least one type of cytopenia, and a majority of them developed a severe cytopenia, consistent with the results reported in previous studies (10, 11). From the perspective of three-lineage kinetics, the occurrence of severe neutropenia peaked earlier than those of severe anemia and severe thrombocytopenia, emphasizing the importance of early infection prevention. Since the immediate effects of lymphodepleting or bridging chemotherapy regimens on the hematopoietic system, previous studies have divided cytopenias after CAR-T-cell infusion into two stages (14). Accordingly, we explored risk factors that may lead to early and late cytopenias through univariate and multivariate analyses.

Our results showed that prior HSCT, and baseline HB were significantly associated with the occurrence of early severe cytopenia. Simultaneously, baseline PLT was an independent adverse factor associated with late thrombocytopenia. This finding indicates the possibility of direct toxicity to the hematopoietic system as a result of earlier treatment (including prior HSCT and numerous cycles of cytotoxic chemotherapy) (20), which may have resulted in inadequate bone marrow function in the presence of hematopoietic stress. Prior research has shown that myeloablative conditioning regimen combined with autologous hematopoietic stem cell transplantation might provide better disease debulking than conventional bridging regimens and thereby lead to greater efficacy of subsequent CAR-T-cell therapy in patients with high-risk LBCL (21). Therefore, for some young patients with better physical fitness, we adopted the myeloablative lymphocyte depletion regimen. Although this regimen could inevitably lead to severe early cytopenia, it had no effect on late cytopenia.

Correlation analysis showed that there was a certain correlation between the grade of CRS and the severity of cytopenia, which highlighted CRS as a common risk factor for early severe cytopenia. This conclusion is consistent with previously reported findings (13, 14). In addition, in our study, inflammatory indicators such as CRP and ferritin were substantially related to early hepatotoxicity, and spikes in cytokines, especially IL-6, were also significantly associated with early severe trilineage cytopenia, the rates of which were significantly elevated in groups with severe CRS in previous reports (22). Likewise, we noted that baseline ferritin, and peak ferritin were significantly associated with late thrombocytopenia in the univariate analysis. Consistent with a recent report that Rejeski and colleagues developed a predictive model to identify biomarkers, including baseline inflammatory markers (CRP and ferritin), to predict hepatotoxicity after CAR-T-cell infusion. According to their findings, high CAR-HEMATOTOX scores were associated with higher incidences of severe thrombocytopenia (16). Notably, although severity of CRS was associated with the early severity of hematologic toxicity, it did not affect hematologic recovery. In addition to the potential predictive value of the above serum markers for cytopenia, previous studies have reported that in lymphoma patients, late cytopenia may be related to the tumor microenvironment. Fried (14) argues that a chemokine critical for B-cell maturation and hematopoietic stem cell migration, stromal derived factor-1 (SDF-1), may be related to late cytopenia. Furthermore, in the ZUMA-1 study, four patients were diagnosed with myelodysplastic syndrome (MDS) after a median of 13.5 months (range 4-26), attributed to previous systemic therapies (23). Therefore, we further assessed bone marrow morphology in five patients with severe hematologic toxicity over 3 months and used an MDS FISH panel to determine whether the patients had cytogenetic abnormalities, and there was no evidence of clonal hematopoiesis of indeterminate potential (CHIP) or early MDS among them. However, this conclusion needs to be confirmed in studies with larger sample sizes and more precise detection methods, such as next-generation sequencing (NGS).

Previous report has shown that CD19/CD22 bispecific CAR-T product had equivalent potency and lower CRS response versus CD19 (24). To reduce the risk of relapse mediated by antigen negative clonal escape, in our institution a considerable proportion of patients were treated with CD19/CD22 bispecific CAR-T cells. Encouragingly, our observations demonstrated that modifications to the CAR-T target did not raise the risk of hematologic toxicity. In addition, according to previous reports (25), CRS cases are more severe with the usage of CARs that employ CD28 as a costimulatory domain compared with the usage of CARs that employ 4-1BB constructs, and Tania Jain’s research showed (26) that the CAR construct was significantly associated with differences in the peak level, expansion, and persistence of various CAR-T-cells, factors that contribute to the development of cytopenias. In our study, the patients all received CAR-T-cells with 4-1BB as the costimulatory domain, and we could include commercial CAR-T-cells with the CD28 construct as a control for further comparative analysis in the future.

Overall, the infection rates after CAR-T-cell therapy appeared to be equivalent to those seen in similarly intensively pretreated patients. Many studies have been conducted to assess infection rates in individuals receiving CAR-T-cell treatment. The incidence of early infection (<30 d) ranges from 18% to 60% (12, 27–29) in prospective clinical trials and retrospective analyses. For example, Hill (12) described 23% of patients with different B lymphoid malignancies had infection during the first 28 days following CAR-T-cell infusion. Park (27)reported that adult B-ALL patients who underwent CD19-28z CAR-T-cell treatment experienced a 40% frequency of infections. The causes of infection in patients who have received CAR-T-cell treatment are multifactorial, including cumulative immunodeficiency brought on by prior therapies and the inherent susceptibility of patients with hematological malignancies to infection (12). The use of lymphodepleting chemotherapy to eliminate immune cells (30) as well as the use of immune-suppressing medicines such as high-dose corticosteroids and tocilizumab to alleviate the side effects of CAR-T-cell therapy may significantly increase the risk of infection (31). Furthermore, CD19 CAR-T-cell products that target B cells may cause B-cell aplasia, as a result, hypoimmunoglobulinemia, which increases the risk of infection. Post-CAR-T-cell cytopenia is also gradually being recognized as an important factor, and this cytopenia is more severe than predicted following chemotherapy and lasts long after CRS has resolved. Our study also suggests that severe cytopenia is associated with an increased incidence of infection and significantly higher rates of using broad-spectrum antibiotics. Consistent with its association with infection, severe cytopenia is also associated with an increased risk of hemorrhage and a significantly increased number of blood transfusions, which indirectly suggests the need of more treatment related cost. Furthermore, cytopenia can also negatively impact CAR-T-cell therapy outcomes. According to Rejeski and colleagues, poorer clinical outcomes were associated with higher CAR-HEMATOTOX scores. In that analysis, the CAR-HEMATOTOX score was found to have a relationship with OS (P=0.09) and PFS (P=0.07) (16). This conclusion is consistent with the results of our survival analysis. In fact, most patients eventually develop disease relapse, and cytopenia jeopardizes the capacity to provide continued treatment after relapse and may limit participation in clinical studies. This factor further contributes to the poorer prognosis of patients.

In view of the above possible causes of severe cytopenia and adverse outcomes, the predictors we explored have instructive significance for the clinical management of cytopenia. These mainly included the following points: 1. Early supportive intervention is required for patients with high-risk baseline characteristics, 2. Potential modifications to lymphodepleting protocols. For example, cyclophosphamide can be replaced by bendamustine (32), And 3. Reasonable application of corticosteroids and tocilizumab for CRS. For the management of late hematologic toxicity, in addition to supportive care, Naman et al. (33) suggest that hematopoietic stem cell rescue is also an appropriate option.

In general, this study has the following limitations. First, this was a single-institution study with a high ratio of CD19/CD22 CARs, limiting its overall applicability. Next, this was a retrospective study with a relatively short follow-up time, and time of blood cells recovery was not traced in all patients. Again, we lacked peripheral blood samples from patients with persistent cytopenia, precluding us from studying other mechanistic reasons for late hematological toxicity. Furthermore, in clinical practice, the use of granulocyte-colony stimulating factor (GCSF), blood transfusions or corticosteroids will inevitably interfere with blood count results. But we can’t get the complete data. This problem will need to be further studied in a prospective manner.

In summary, our study provides clinical evidence for the hypothesis that CRS and CRS-related cytokines (eg. IL-6, CRP and ferritin) are associated with the severity of early cytopenia. Myelotoxic conditioning regimen, prior HSCT, and baseline HB were independent adverse factors associated with the occurrence of early severe cytopenia. Furthermore, baseline PLT count was an independent factor associated with late thrombocytopenia after CAR-T-cell therapy. Our data suggest that clinicians should focus on cytopenia and guide the accurate prediction and appropriate management of post-CAR-T-cell therapy cytopenia.

## Data availability statement

The raw data supporting the conclusions of this article will be made available by the authors, without undue reservation.

## Ethics statement

The studies involving human participants were reviewed and approved by the Institutional Ethics Committee of the First Affiliated Hospital of Suzhou University. The patients/participants provided their written informed consent to participate in this study. Written informed consent was obtained from the individual(s) for the publication of any potentially identifiable images or data included in this article.

## Author contributions

DW, CL, and YX contributed to the conception of the study and manuscript revision. HG, JL, and GC contributed to data collection. LY designed and manufactured the CAR-T-cells; JZ, YZ, MS, and XZ analyzed data and wrote the manuscript. All authors contributed to the article and approved the submitted version.

## Funding

National Natural Science Foundation of China (82020108003), National Natural Science Foundation of China (81730003), National Key R&D Program of China (2019YFC0840604, 2017YFA0104502), Key R&D Program of Jiangsu Province (BE2019798), Priority Academic Program Development of Jiangsu Higher Education Institutions (PAPD), Jiangsu Medical Outstanding Talents Project (JCRCA2016002), Jiangsu Provincial Key Medical Center (YXZXA2016002), Suzhou Science and Technology Program Project (SLT201911).

## Conflict of interest

The authors declare that the research was conducted in the absence of any commercial or financial relationships that could be construed as a potential conflict of interest.

## Publisher’s note

All claims expressed in this article are solely those of the authors and do not necessarily represent those of their affiliated organizations, or those of the publisher, the editors and the reviewers. Any product that may be evaluated in this article, or claim that may be made by its manufacturer, is not guaranteed or endorsed by the publisher.
