# Supplementary material for: Cytopenia after chimeric antigen receptor T cell immunotherapy in relapsed or refractory lymphoma
Source: Front Immunol. 2022 Sep 5;13:997589. doi: 10.3389/fimmu.2022.997589 (PMC9484486; doi:10.3389/fimmu.2022.997589)
Supplement: Supplementary file 1 [file DataSheet_1.docx]

**Supplementary Materials**

**Supplement Table 1. Univariate Analysis of Factors Associated with the incidence of early severe neutropenia**

|  | **p-value** | **OR** | **95% CI** |
| --- | --- | --- | --- |
| **Age** | 0.444 | 0.989 | 0.960-1.018 |
| **Sex (M/F)** | 0.964 | 1.017 | 0.485-2.136 |
| **Disease Type** |  |  |  |
| **Aggressive B-cell lymphoma^a^** | 1^c^ |  |  |
| **Indolent B-cell lymphoma^b^** | 0.192 | 5.059 | 0.444-57.639 |
| **AITL** | 0.810 | 0.667 | 0.025-18.059 |
| **HL** | 0.355 | 4.000 | 0.211-75.659 |
| **Ann Arbor Stage (≤II/>II)** | 0.137 | 0.312 | 0.067-1.450 |
| **Bone marrow involvement (yes/no)** | 0.655 | 0.839 | 0.388-1.813 |
| **lines of prior therapy (≤2/>2)** | 0.243 | 0.640 | 0.303-1.353 |
| **Response before treatmen (PR/PD,SD)** | 0.381 | 0.711 | 0.331-1.524 |
| **Prior HSCT (yes/no)** | 0.042 | 0.267 | 0.075-0.953 |
| **LDH** | 0.217 | 1.001 | 0.999-1.003 |
| **Lymphodepleting chemotherapy (FC/SEAM)** | 0.091 | 6.000 | 0.753-47.787 |
| **CAR Product** |  |  |  |
| **CD19** | 1^d^ |  |  |
| **CD19/CD22** | 0.960 | 0.955 | 0.156-5.846 |
| **CD19,CD20** | 0.679 | 1.421 | 0.269-7.508 |
| **CD30** | 0.691 | 1.500 | 0.203-11.088 |
| **Baseline NE** | 0.965 | 1.002 | 0.898-1.119 |
| **Baseline HB** | 0.029 | 0.981 | 0.964-0.998 |
| **Baseline PLT** | 0.847 | 1.000 | 0.997-1.004 |
| **Baseline CRP** | 0.198 | 1.010 | 0.995-1.025 |
| **Baseline ferritin** | 0.051 | 1.001 | 1.000-1.001 |

Aggressive B-cell lymphoma^a^ include DLBCL, PMBCL, GZL, Transformed Lymphoma, MCL, BL

Indolent B-cell lymphoma^b^ include MZL, CLL, FL

Aggressive B-cell lymphoma^c^ group was defined as the control group

CD19^d^ group was defined as the control group

**Supplement Table 2. Univariate Analysis of Factors Associated with the incidence of early severe thrombocytopenia**

|  | **p-value** | **OR** | **95% CI** |
| --- | --- | --- | --- |
| **Age** | 0.369 | 0.988 | 0.961-1.015 |
| **Sex (M/F)** | 0.606 | 1.202 | 0.598-2.413 |

| **Disease Type** |  |  |  |
| --- | --- | --- | --- |
| **Aggressive B-cell lymphoma^a^** | 1^a^ |  |  |
| **Indolent B-cell lymphoma^b^** | 0.406 | 0.357 | 0.032-4.048 |
| **AITL** | 0.999 | 0.000 | 0.000-0.000 |
| **HL** | 0.355 | 0.250 | 0.013-4.729 |

| **Ann Arbor Stage (≤II/>II)** | 0.612 | 0.757 | 0.257-2.226 |
| --- | --- | --- | --- |
| **Bone marrow involvement(yes/no)** | 0.398 | 0.726 | 0.346-1.525 |
| **lines of prior therapy (≤2/>2)** | 0.177 | 0.618 | 0.308-1.242 |
| **Response before treatment (PR/PD,SD)** | 0.369 | 0.718 | 0.349-1.479 |
| **Prior HSCT (yes/no)** | 0.002 | 4726 | 1.800-12.409 |
| **LDH** | 0.016 | 1.002 | 1.000-1.004 |
| **Lymphodepleting chemotherapy (FC/SEAM)** | 0.003 | 22.286 | 2.800-177.353 |

| **CAR Product** |  |  |  |
| --- | --- | --- | --- |
| **CD19** | 1^d^ |  |  |
| **CD19/CD22** | 0.874 | 0.877 | 0.173-4.447 |
| **CD19,CD20** | 0.722 | 1.312 | 0.294-5.850 |
| **CD30** | 0.848 | 0.833 | 0.129-5.396 |

| **Baseline NE** | 0.803 | 1.013 | 0.915-1.121 |
| --- | --- | --- | --- |
| **Baseline HB** | 0.000 | 0.968 | 0.950-0.985 |
| **Baseline PLT** | 0.870 | 1.000 | 0.996-1.003 |
| **Baseline CRP** | 0.006 | 1.020 | 1.003-1.034 |
| **Baseline ferritin** | 0.023 | 1.000 | 1.000-1.001 |

Aggressive B-cell lymphoma^a^ include DLBCL, PMBCL, GZL, Transformed Lymphoma, MCL, BL

Indolent B-cell lymphoma^b^ include MZL, CLL, FL

Aggressive B-cell lymphoma^c^ group was defined as the control group

CD19^d^ group was defined as the control group

**Supplement Table 3. Multivariable Analysis of Factors Associated with the incidence of early severe neutropenia**

|  | **p-value** | **OR** | **95% CI** |
| --- | --- | --- | --- |
| **Prior HSCT (yes/no)** | 0.031 | 4.174 | 1.138-15.310 |
| **Baseline HB** | 0.021 | 0.979 | 0.962-0.997 |

**Supplement Table 4. Univariate Analysis of Factors Associated with the late neutropenia**

| **Variable** | **Univariable** |  | **Multivariable** |  |
| --- | --- | --- | --- | --- |
|  | **Hazard ratio (95%CI)** | **P** | **Hazard ratio (95%CI)** | **P** |
| **Age** | 0.996(0.959-1.036) | 0.852 |  |  |
| **Sex** | 0.591(0.223-1.563) | 0.289 |  |  |
| **Disease Type** |  |  |  |  |
| **Aggressive B-cell lymphoma^a^** | 1^c^ |  |  |  |
| **Indolent B-cell lymphoma^b^** | 3.461E+8(0.000-) | 0.999 |  |  |
| **AITL** | 1.000(0.000-) | 1.000 |  |  |
| **HL** | 2.610E+18(0.000-) | 0.999 |  |  |
| **Ann Arbor Stage (≤2/>2)** | 0.828(0.169-4.064) | 0.816 |  |  |
| **Bone marrow involvement(yes/no)** | 0.528(0.177-1.577) | 0.253 |  |  |
| **lines of prior therapy (≤2/>2)** | 1.533(0.574-4.094) | 0.394 |  |  |
| **Prior HSCT (yes/no)** | 1.786(0.603-5.290) | 0.295 |  |  |
| **Response before treatment (PR/SD,PD)** | 0.991(0.370-2.657) | 0.986 |  |  |
| **Pre-LD NE** | 0.711(0.476-1.060) | 0.094 |  |  |
| **Pre-LD HB** | 0.981(0.958-1.005) | 0.128 |  |  |
| **Pre-LD plt** | 0.994(0.986-1.001) | 0.106 |  |  |
| **LDH** | 1.000(0.998-1.002) | 0.740 |  |  |
| **Lymphodepleting chemotherapy (FC/SEAM)** | 0.355(0.043-2.902) | 0.334 |  |  |
| **Baseline CRP** | 1.004（0.986-1.021） | 0.680 |  |  |
| **CAR Product** |  |  |  |  |
| **CD19** | 1^d^ |  |  |  |
| **CD19/CD22** | 0.556(0.078-3.965) | 0.558 |  |  |
| **CD19, CD20** | 0.317(0.051-1.957) | 0.218 |  |  |
| **CD30** | 0.750(0.087-6.468) | 0.794 |  |  |
| **Baseline ferritin** | 1.000（1.000-1.001） | 0.296 |  |  |
| **CRS grade (<2/≥2)** | 0.537（0.144-2.000） | 0.354 |  |  |
| **CRP max** | 0.999（0.995-1.003） | 0.516 |  |  |
| **Ferritin max** | 1.000（1.000-1.000） | 0.721 |  |  |
| **IL2 max** | 1.013（0.988-1.039） | 0.297 |  |  |
| **IL4 max** | 0.980（0.929-1.034） | 0.452 |  |  |
| **IL6 max** | 1.000（1.000-1.001） | 0.085 |  |  |
| **IL10 max** | 1.000（0.994-1.006） | 0.972 |  |  |
| **TNFɑ max** | 0.976（0.930-1.024） | 0.316 |  |  |
| **IFNγmax** | 0.998（0.993-1.004） | 0.557 |  |  |
| **IL17 max** | 0.996（0.980-1.013） | 0.645 |  |  |

Aggressive B-cell lymphoma^a^ include DLBCL, PMBCL, GZL, Transformed Lymphoma, MCL, BL

Indolent B-cell lymphoma^b^ include MZL, CLL, FL

Aggressive B-cell lymphoma^c^ group was defined as the control group

CD19^d^ group was defined as the control group
